# Supplementary material for: Long-Term Effects of a Stepwise, Multimodal, Non-Restrictive Antimicrobial Stewardship Programme for Reducing Broad-Spectrum Antibiotic Use in the ICU
Source: Antibiotics (Basel). 2024 Jan 29;13(2):132. doi: 10.3390/antibiotics13020132 (PMC10886276; doi:10.3390/antibiotics13020132)
Supplement: Supplementary file 1 [file antibiotics-13-00132-s001.zip › antibiotics-2808327-supplementary.pdf]

SUPPLEMENTARY APPENDIX

TABLE S1. Characteristics and outcomes of patients in the ICU. Period 2011-2019

TABLE S2. Use of antibiotic classes by DDD/100 patient-days in the ICU. Period 2011-2019

TABLE S3. Point prevalence survey characteristics. Period 2013-2019

Table S1. Characteristics and outcomes of patients in the ICU. Period 2011-2019

Year	***Total admitted ICU patients*** *N*	***Age (mean ± SD)***	***APACHE II (mean ± SD)***	***Length of stay (mean ± SD)***	***ICU Mortality (%)***	***30-day readmission (%)***
2011	1,453	62±14	16±8	8.6±13	23	5.0
2012	1,253	63±13	17±8	9.6±15	24	7.4
2013	1,259	63±13	17±8	9.4±15	21	5.6
2014	1,284	64±13	17±8	9.0±13	19	4.8
2015	1,362	63±14	15±8	8.7±16	20	7.1
2016	1,500	66±14	16±8	7.5±11	21	5.4
2017	1,517	63±14	16±7	7.5±10	18	5.5
2018	1,448	64±14	15±9	7.7±10	19	6.8
2019	1,390	63±14	16±8	8.0±10	18	6.0

Footnotes: Aggregated data from the hospital's standard reporting system

Table S2. Use of antibiotic classes by DDD/100 patient-days in the ICU. Period 2011-2019

Year	Period	ATM	AB	PEN	CEPH	CARB	FQ	BSA	AMG	GP	Cs	MC	TG	CLD+MTZ	DPT
2011	1	174.7	158.1	55.0	12.3	13.5	13.0	31.4	3.9	12.1	19.6	4.4	0.2	6.2	4.0
	2	194.5	176.4	51.6	18.7	20.4	14.5	43.7	2.2	13.6	29.9	7.1	0.1	5.7	4.3
	3	243.1	221.7	61.9	12.4	30.3	19.0	48.4	5.7	16.8	47.9	3.5	1.0	6.9	3.7
2012	1	180.8	159.7	45.2	15.5	16.3	16.1	37.5	3.0	18.2	27.6	6.5	0.3	3.3	1.1
	2	201.6	176.6	47.8	19.3	20.0	17.6	38.5	3.2	15.7	30.0	0.7	0.1	7.4	4.5
	3	211.2	182.5	55.3	14.3	24.5	16.4	38.0	1.9	21.5	30.4	4.6	1.0	2.5	2.2
2013	1	170.4	151.7	41.7	17.5	19.0	13.3	39.7	2.5	12.7	25.5	3.6	0.2	5.1	3.5
	2	169.9	152.0	36.1	19.1	18.7	14.0	42.7	1.1	16.3	26.2	4.7	0.2	3.7	2.7
	3	173.0	161.3	45.5	13.7	26.5	18.0	42.1	2.0	17.3	20.4	4.8	0.1	3.3	1.8
2014	1	189.0	165.4	54.6	15.7	22.1	13.1	34.1	1.9	17.6	19.7	3.6	0.5	3.5	5.0
	2	184.7	166.5	47.2	14.4	21.8	14.4	43.2	1.5	24.7	20.8	2.0	0.8	5.1	6.9
	3	199.2	180.8	46.2	15.8	30.4	17.0	47.0	1.7	22.9	17.0	6.5	1.2	7.0	6.0
2015	1	191.3	168.3	41.2	17.7	26.3	18.1	44.2	1.6	18.7	19.1	4.0	0.8	4.5	7.5
	2	189.4	167.2	45.6	17.8	27.8	13.3	47.2	1.4	16.6	18.2	5.2	0.9	3.7	5.3
	3	203.5	174.0	39.2	17.2	36.2	17.3	59.6	1.7	23.9	20.0	3.1	0.8	3.8	2.8
2016	1	195.4	174.5	39.1	26.2	24.9	15.8	53.0	0.8	18.9	26.4	4.2	1.5	4.4	5.7
	2	193.9	166.0	50.1	20.0	24.3	19.3	43.1	3.1	14.3	10.3	5.0	2.2	3.6	6.0
	3	159.8	142.4	35.3	18.8	19.3	16.0	37.0	0.8	16.0	14.6	5.5	0.9	2.1	5.1
2017	1	155.7	133.1	27.5	25.8	17.6	20.6	31.7	2.2	11.9	10.8	1.3	2.4	1.8	4.9
	2	146.0	132.5	27.2	21.1	25.3	17.6	42.2	1.3	13.4	4.8	3.2	1.7	2.1	7.3
	3	191.3	159.5	38.6	26.8	20.1	23.0	38.5	0.9	9.2	11.2	3.1	2.1	1.5	10.4
2018	1	174.0	148.6	34.8	19.2	21.1	13.4	36.0	1.2	16.7	6.1	3.8	4.2	6.1	11.7
	2	166.6	144.9	38.2	21.0	19.3	12.4	36.5	0.4	17.4	4.3	3.3	2.3	4.3	17.3
	3	142.1	127.1	36.8	14.2	24.2	10.2	43.2	1.5	19.3	2.1	4.3	0.1	2.6	5.5
2019	1	152.4	129.8	44.8	16.0	14.7	14.5	35.1	0.9	14.1	2.9	2.6	0.5	1.9	8.0
	2	158.9	138.4	35.9	18.2	18.9	12.4	34.6	1.6	20.0	4.3	6.3	2.1	3.0	7.8
	3	182.7	157.4	38.1	15.5	18.6	12.5	39.9	1.1	18.1	2.8	6.2	1.0	1.9	15.0

Footnotes: Table shows main classes of antibiotics in use during the study. Period: Consumption was collected on a 4-monthly basis. It includes three periods per year; ATM: Antimicrobials; AB: Antibiotics; PEN: Penicillins (cloxacillin, penicillin G, ampicillin, amoxicillin-clavulanate, and piperacillin-tazobactam); CEPH: Cephalosporins (cefuroxime, ceftriaxone, ceftazidime, and cefepime); CARB: Carbapenems (meropenem, imipenem, and ertapenem); FQ: Fluoroquinolones (ciprofloxacin, levofloxacin, and moxifloxacin); BSA: Broad-spectrum antibiotics (meropenem, imipenem, piperacillin-tazobactam, and cefepime); AMG: Aminoglycosides (amikacin, gentamicin, and tobramicin); GP: Glycopeptides (teicoplanin and vancomycin); Cs: Colistin; MC: Macrolides (azithromycin, clarithromycin, and erithromycin); TG: Tigecycline; CLD+MTZ: Clindamicin + metronidazole; DPT: Daptomycin.

Table S3. Point prevalence survey characteristics. Period 2013-2019

	2013	*2014*	*2015*	*2016*	*2017*	*2018*	*2019*	*P-Value*
Overall patients included in the point prevalence survey, N	**360**	**346**	**353**	**378**	**389**	**359**	**388**	
Patients on antimicrobial therapy, N (%)	248(69)	243 (70)	262 (74)	280 (74)	264 (68)	257 (72)	284 (73)	0.01
Overall antimicrobials on use, N	**383**	**396**	**430**	**470**	**476**	**450**	**493**	
Correct duration of therapy, N (%)	320(84)	301(76)	354 (82)	371 (79)	405 (85)	383 (85)	429 (87)	0.17
Correct de-escalation, N (%)	320(84)	321(81)	384 (89)	421 (90)	444 (93)	406 (90)	458 (93)	<0.01
Appropriateness of therapy, N (%)	291(76)	278 (70)	335 (78)	354 (75)	398 (84)	359 (80)	422 86)	<0.01

Footnotes: P-Value was assessed using Pearson's Chi-squared test
